# Sex Disparity in Myopia Explained by Puberty Among Chinese Adolescents From 1995 to 2014: A Nationwide Cross-Sectional Study

**DOI:** 10.3389/fpubh.2022.833960

**Published:** 2022-05-30

**Authors:** Rongbin Xu, Panliang Zhong, Catherine Jan, Yi Song, Xiuqin Xiong, Dongmei Luo, Yanhui Dong, Jun Ma, Randall S. Stafford

**Affiliations:** ^1^School of Public Health, Institute of Child and Adolescent Health, Peking University, Beijing, China; ^2^School of Public Health and Preventive Medicine, Monash University, Melbourne, VIC, Australia; ^3^Lost Child's Vision Project, Sydney, NSW, Australia; ^4^Centre for Eye Research Australia, University of Melbourne, Melbourne, VIC, Australia; ^5^Department of Ophthalmology and Surgery, Faculty of Medicine, Dentistry & Health Sciences, University of Melbourne, Melbourne, VIC, Australia; ^6^Centre for Health Policy, Melbourne School of Population and Global Health, The University of Melbourne, Parkville, VIC, Australia; ^7^Department of Medicine, Stanford Prevention Research Center, Stanford University School of Medicine, Stanford, CA, United States

**Keywords:** puberty, myopia, sex disparity, adolescents, Chinese, menarche, spermarche

## Abstract

**Importance:**

Girls in East Asia have a higher myopia prevalence than boys. Less research has been done on whether girls' earlier puberty could explain this sex difference.

**Objective:**

The purpose of this study was to evaluate the association between myopia and puberty and the role of puberty in explaining the sex disparity in adolescent myopia prevalence.

**Design, Setting, and Participants:**

In this nationwide cross-sectional study, data came from five consecutive national surveys from 1995 to 2014 in China. We included 338,896 boys aged 11–18 and 439,481 girls aged 9–18.

**Main Outcomes and Measures:**

Myopia was defined according to unaided distance visual acuity and subjective refraction; puberty status was defined dichotomously as menarche or spermarche status. The association between myopia and puberty was evaluated by robust Poisson GEE regression. Mediation analyses were used to quantify how much of the sex disparity in myopia could be explained by puberty.

**Results:**

Post-menarche girls and post-spermarche boys showed 29–41% and 8–19% higher risk of myopia than pre-menarche girls and pre-spermarche boys, respectively. The association remained significant in girls [prevalence ratio (PR) = 1.07, 95%CI:1.04–1.10] but disappeared in boys (*p* > 0.05) after adjusting for potential confounders. Girls had a 12–23% higher risk of myopia than boys. A total of 16.7% of the sex disparity in myopia could be explained by girls' earlier puberty, whereas 11.1% could be explained by behavioral factors.

**Conclusion and Relevance:**

Puberty status is independently associated with myopia in girls but not in boys. A significant proportion of the sex disparity in adolescent myopia could be explained by girls' earlier puberty, suggesting the need to consider sex-differentiated strategies for myopia prevention and treatment.

## Introduction

Myopia (near-sighted vision) has emerged as a major global public health concern (1) with its rapidly increasing prevalence (2) and heavy economic burden (3). The higher prevalence of myopia in girls is a consistent phenomenon in most ethnicities, but this trend has few satisfactory explanations (2). East Asians, including the Chinese (4), have the highest prevalence of myopia worldwide, reaching 80% at the age of 18 years. We previously found that the earlier a girl enters puberty, the higher their risk of myopia (5). Given that girls usually enter puberty 1–2 years earlier than boys (6, 7), earlier puberty may partly explain the higher prevalence of myopia in girls.

A cohort study found that nearly 80% of new myopia cases occurred in individuals aged 9–13 years (8), suggesting that myopia mostly develops during early and mid-puberty (1). Thus, we hypothesize that puberty development might be associated with myopia onset. This hypothesis is further supported by the finding that boys and girls with earlier growth spurts also experienced earlier axial growth and myopia onset (9). A total of two other cohort studies showed that growth in height before age 10 contributed was not associated with myopia development, indicating that puberty development after age 10 may play a bigger role (10, 11).

Evidence using puberty indicators other than growth is controversial and limited. A number of two cross-sectional studies investigating adults in India and South Korea found that women with an earlier menarche age had a higher prevalence of myopia. These results, however, may be compromised by recall bias because the age of menarche- and myopia-related covariates was collected in adulthood (12, 13). In contrast, another study found no association between the age of menarche and the age of axial growth or myopia onset, but this negative finding may result from selection bias and low statistical power because it only included 1,779 children from 3 schools in Singapore (9). Furthermore, studies have not been able to establish an association between myopia and spermarche, the male-specific puberty indicator. More studies with a large sample size and good measures of puberty status during adolescence are needed to clarify the role of puberty in myopia development. Such studies may be especially critical for China, the country with the largest myopic population (4).

Based on previous studies, we hypothesize that adolescent myopia is positively associated with the onset of puberty, as represented by menarche or spermarche status, and this association may help explain girls' higher myopia prevalence in China. The Chinese National Survey on Students' Constitute and Health (CNSSCH), a national survey of school-aged children, provided us with data to approach these questions. Testing these hypotheses could lead to more targeted or sex-specific strategies to prevent myopia.

## Methods

### Study Design and Participants

Data were extracted from the 1995, 2000, 2005, 2010, and 2014 cycles of the CNSSCH, a series of cross-sectional national surveys among school-aged children in China that used identical stratified random cluster sampling procedures in each cycle. In total, the surveys reached 1,081,956 Han ethnicity students (the dominant ethnic group in China) aged 7–18, of which 1,080,030 (99.8%) had data on myopia. The CNSSCH covered 30 of the 31 mainland provinces (4 municipalities were also treated as provincial units), excluding Tibet where the Han people are a minority. Children from recognized non-Han ethnicity minority groups were not included (these groups constitute 8.9% of the population of the 30 included provinces). In each province, three cities or regions at different levels of economic development or regional socioeconomic status (SES) (“upper,” “moderate,” and “low”) were chosen. Children aged 7–18 clustered by classroom were randomly chosen from these schools, ensuring that each sex × age combination in each city/region included at least 100 children (6). The project was approved by the Medical Research Ethics Committee of Peking University Health Science Center (IRB00001052-18002).

### Visual Acuity and Refraction Status Measurements

Myopia was defined based on the vision chart assessment of unaided distance visual acuity (VA) (4) in the worse eye combined with simple subjective refraction. Unaided distance VA for each eye was measured by certified optometrists using a retro illuminated logarithm of the minimum angle of resolution (logMAR) chart with tumbling-E optotypes (Precision Vision, Denver Colorado) (8). Reduced VA was defined as distance VA worse than 6/6.

For eyes with reduced VA, subjective refraction was used to detect the refractive status with a positive/ negative diopter spherical lens of +/-0.75D. Compared with the unaided distance VA, if the distance VA wearing the positive lens reduced ≥1 line on the chart, and the distance VA wearing the negative lens improved ≥1 line, then the examined eye was defined as having “myopia”; if the result was reversed, then the examined eye was defined as having “hyperopia.” Any other situations were defined as “other reduced VA.” If one of the two eyes was defined as myopia, then the participant was defined as having myopia. According to a validation trial performed by our collaborators in 2012 (refer to Supplementary Material), our definition of myopia achieved a sensitivity of 91.9% and a specificity of 83.6%, compared with the most commonly used definition (3) (spherical equivalent refractive error measured by cycloplegic refraction ≤-0.50 D).

### Puberty Status Measurements

In each CNSSCH, individual puberty status was defined by the menarche or spermarche status responses given to sex-matched interviewers (6). Girls aged ≥9 years were asked whether menarche had occurred by a school nurse or female physician (6). Similarly, boys ≥11 years were asked whether they had experienced a first ejaculation by male physicians or health professionals (5). As detailed in our previous publications, when needed we used several scripted statements from our well-trained interviewers to ensure that students understood the question and answered the question in a relaxed way (5, 6).

### Other Measurements

Participants in the 2014 CNSSCH were asked to complete a self-administered questionnaire in their classrooms and under the guidance of trained investigators. The questionnaire was designed by a panel of experts. Pilot studies were carried out to test whether the questionnaire could be understood and answered accurately by the students. Prior to filling in the questionnaire, students were informed that their answers would be kept confidential and would have no effect on their grades. The questionnaire covered different behaviors, such as sleep duration, physical activity, homework time, near screen time, weekend outdoor activity, and weekend study activity. For individual students, weekend outdoor activity and weekend study activity were classified as “in top 3” and “not in top 3,” meaning that the outdoor (or study) activity is one of the top three choices that the participants do on weekends. Age in years and age in days (exact age, presented in hundreds) were both calculated according to participants' date of birth and date of physical examination in the survey. Provincial Gross Domestic Product (GDP) per capita at 2014 prices in different survey years was sourced from the China Statistical Yearbook to provide a measure of regional socioeconomic status (SES). For each participant, VA, puberty status, and all other measures were performed in 1 day.

### Statistical Analyses

First, we used the full sample to evaluate the association between myopia and puberty status. We compared the age-standardized prevalence of myopia between pre-menarche/spermarche and post-menarche/spermarche girls and boys across different ages and survey years using chi-square tests. A total of 338,896 boys aged 11–18 and 439,481 girls aged 9–18 with complete data on myopia and puberty status were included in this analysis. We used robust Poisson regression models based on a generalized estimated equation (Poisson GEE) to detect the association between myopia and puberty status (14, 15). This family of models adjusts for the cluster effect of school and estimates prevalence ratios (PRs), which are unbiased estimators of relative risk in cross-sectional studies (14, 15). PRs avoid the problem of odds ratios, which overestimate the relative risk when the prevalence is higher than 10% (14, 15).

Second, we used matched samples to evaluate the association between myopia and puberty status. To make the pre- and post-menarche girls or pre- and post-spermarche boys as comparable as possible, we extracted 5,641 pairs of boys and 6,151 pairs of girls from the 2014 CNSSCH. In each pair, one was pre-menarche or pre-spermarche whereas the other was post-menarche or post-spermarche, and they were the same age and from the same school. This pairing procedure helped to control the effect of age and other confounders at the school level or above and avoids the multi-collinearity of adding age to the regression model.

Finally, mediation analyses with two steps of regression were used to estimate the proportion of sex disparity in myopia explained by puberty and myopia-related behaviors, quantified as the percentage of excess risk mediated (PERM) (refer to Supplementary Material) (16). The median age at menarche or spermarche and their 95% CI were estimated by probit analyses (5, 6). The PRs and their 95% CI were estimated for each model. A two-sided *p* < 0.05 was considered statistically significant. We used SPSS (version 20.0, IBM, Chicago, Illinois, USA) to perform the probit analyses. All other analyses were performed in R (version 3.3.2, Boston, Massachusetts), and the *geepack* package (version 1.2-1) in R was used to perform the regression analyses.

## Results

### The Myopia Prevalence Among Pre- and Post- Menarche/Spermarche Girls and Boys

From 1995 to 2014, the age-standardized prevalence of myopia was 8.7–12.8% points greater in post-menarche girls than pre-menarche girls aged 9–18 (all *p* < 0.001).

A similar pattern was seen in boys, but the disparity between pre- and post-puberty boys was smaller than that of girls. From 1995 to 2014, the age-standardized prevalence of myopia was only 2.5–5.8% points greater in post-spermarche boys than pre-spermarche boys aged 11–18 (all *p* < 0.001) (Table 1).

**Table 1:** Comparison of myopia prevalence between pre- and post-menarche/spermarche subjects by sex and age, 1995–2014 [sample size (%)].

	**1995**			**2000**			**2005**			**2010**			**2014**		
**Age (years)**	**Pre**	**Post**	***p*-Value**	**Pre**	**Post**	***p*-Value**	**Pre**	**Post**	***p*-Value**	**Pre**	**Post**	***P*-Value**	**Pre**	**Post**	***P*-Value**
**Girls**															
9	8,179 (18.6)	15 (33.3)	0.14	8,912 (17.8)	87 (32.2)	<0.001	8,954 (28.3)	43 (41.9)	0.05	7,448 (38.3)	53 (45.3)	0.30	7,487 (39.9)	84 (44.0)	0.45
10	8,679 (22.7)	80 (27.5)	0.31	8,942 (22.7)	1,78 (27.5)	0.13	8,945 (35.2)	272 (41.2)	0.04	7,849 (46.3)	235 (58.7)	<0.001	7,546 (47.7)	536 (54.1)	0.01
11	7,988 (28.7)	676 (40.2)	<0.001	8,088 (27.3)	874 (38.0)	<0.001	8,017 (40.4)	1,286 (50.0)	<0.001	7,005 (52.4)	1,490 (62.8)	<0.001	6,211 (54.1)	2,233 (63.5)	<0.001
12	5,924 (34.0)	2,675 (43.7)	<0.001	5,848 (29.4)	3,170 (43.4)	<0.001	5,824 (43.1)	3,311 (54.6)	<0.001	4,238 (54.7)	4,423 (60.7)	<0.001	3,514 (56.2)	5,178 (64.7)	<0.001
13	2,458 (40.6)	6,096 (52.3)	<0.001	2,583 (34.4)	6,377 (46.5)	<0.001	2,360 (46.9)	6,976 (57.5)	<0.001	1,613 (56.1)	7,123 (68.8)	<0.001	1,139 (62.0)	7,688 (70.3)	<0.001
14	808 (42.3)	7,767 (56.2)	<0.001	897 (42.3)	8,071 (53.9)	<0.001	688 (47.4)	8,586 (63.6)	<0.001	374 (60.7)	8,350 (71.7)	<0.001	266 (63.5)	8,607 (75.1)	<0.001
15	307 (42.3)	8,277 (61.6)	<0.001	279 (49.5)	8,641 (64.3)	<0.001	203 (63.5)	9,262 (70.0)	0.05	96 (74.0)	8,690 (74.9)	0.76	48 (66.7)	8,878 (76.9)	0.12
16	177 (54.8)	8,340 (68.2)	<0.001	101 (64.4)	8,867 (72.1)	0.08	31 (51.6)	9,354 (75.3)	0.002	45 (71.1)	8,778 (80.3)	0.09	12 (58.3)	8,919 (79.3)	0.06
17	144 (54.9)	8,386 (72.4)	<0.001	152 (66.4)	8,809 (77.3)	0.002	77 (63.6)	9,267 (78.8)	0.001	17 (64.7)	8,849 (82.4)	0.05	11 (81.8)	8,945 (80.0)	0.94
18	114 (62.3)	8,343 (74.0)	0.01	115 (72.2)	8,885 (78.5)	0.10	69 (85.5)	9,304 (79.8)	0.24	18 (66.7)	8,822 (81.6)	0.09	7 (71.4)	8,518 (81.3)	0.46
Total	34,778 (27.4)	50,655 (63.3)	<0.001	35,917 (25.6)	53,959 (64.6)	<0.001	35,168 (37.3)	57,661 (69.9)	<0.001	28,703 (47.9)	56,813 (75.2)	<0.001	26,241 (49.0)	59,586 (75.4)	<0.001
Standardized^*^ total	34,778 (40.1)	50,655 (52.9)	<0.001	35,917 (42.6)	53,959 (53.4)	<0.001	35,168 (50.6)	57,661 (61.3)	<0.001	28,703 (58.5)	56,813 (68.7)	<0.001	26,241 (60.2)	59,586 (68.9)	<0.001
**Boys**															
11	8,605 (22.9)	145 (27.6)	0.22	8,612 (20.8)	275 (14.5)	0.02	8,103 (31.9)	362 (37.3)	0.04	6,872 (45.1)	230 (50.0)	0.16	6,695 (49.2)	360 (50.8)	0.59
12	8,306 (28.5)	434 (29.7)	0.62	8,358 (25.6)	621 (27.7)	0.26	7,248 (35.4)	859 (40.6)	0.003	6,369 (49.6)	801 (47.8)	0.31	6,249 (53.5)	1,133 (55.3)	0.37
13	6,712 (38.5)	2,029 (42.1)	0.004	6,551 (32.2)	2,339 (39.5)	<0.001	5,412 (42.8)	2,592 (47.0)	<0.001	4,935 (55.2)	2,465 (59.0)	0.002	4,506 (57.7)	3,199 (62.1)	<0.001
14	4,330 (41.4)	4,419 (48.3)	<0.001	3,977 (37.5)	5,007 (46.2)	<0.001	2,978 (44.6)	5,373 (52.0)	<0.001	2,596 (58.9)	5,105 (63.7)	<0.001	2,316 (61.4)	5,630 (63.4)	0.19
15	2,238 (45.6)	6,508 (52.7)	<0.001	1,648 (42.1)	7,362 (54.1)	<0.001	1,422 (54.1)	7,679 (59.7)	<0.001	1,181 (62.1)	6,862 (66.9)	<0.001	889 (66.0)	7,393 (70.0)	0.02
16	901 (51.6)	7,781 (61.0)	<0.001	615 (56.6)	8,379 (62.1)	0.01	686 (68.1)	8,630 (67.1)	0.61	382 (74.3)	8,163 (72.4)	0.49	307 (74.3)	8,302 (72.5)	0.77
17	364 (57.1)	8,365 (64.4)	0.01	407 (54.1)	8,487 (68.6)	<0.001	533 (67.7)	8,805 (71.3)	0.09	193 (73.1)	8,480 (74.7)	0.61	174 (74.1)	8,539 (73.8)	0.99
18	286 (63.3)	8,369 (64.6)	0.70	194 (67.0)	8,736 (69.6)	0.49	397 (63.5)	9,051 (71.1)	0.001	183 (71.0)	8,518 (75.2)	0.22	125 (71.2)	8,254 (74.2)	0.40
Total	31,742 (33.4)	38,050 (58.1)	<0.001	30,362 (29.4)	41,206 (59.5)	<0.001	26,779 (39.7)	43,351 (63.6)	<0.001	22,711 (52.0)	40,624 (70.0)	<0.001	21,261 (55.0)	42,810 (70.1)	<0.001
Standardized^*^ total	31,742 (43.6)	38,050 (48.8)	<0.001	30,362 (42.0)	41,206 (47.8)	<0.001	26,779 (51.0)	43,351 (55.7)	<0.001	22,711 (61.2)	40,624 (63.7)	<0.001	21,261 (63.4)	42,810 (65.3)	<0.001

* *Standardized by age, with this aggregate based on each age having the same weight. p-Values for standardized total were obtained from logistic regression models, which tested statistical significance of the effect of puberty status (independent variable) on myopia (dependent variable) after adjusting for age. All other p-values came from a Chi-square test*.

### The Association Between Myopia and Puberty Status

**Figure 1:**
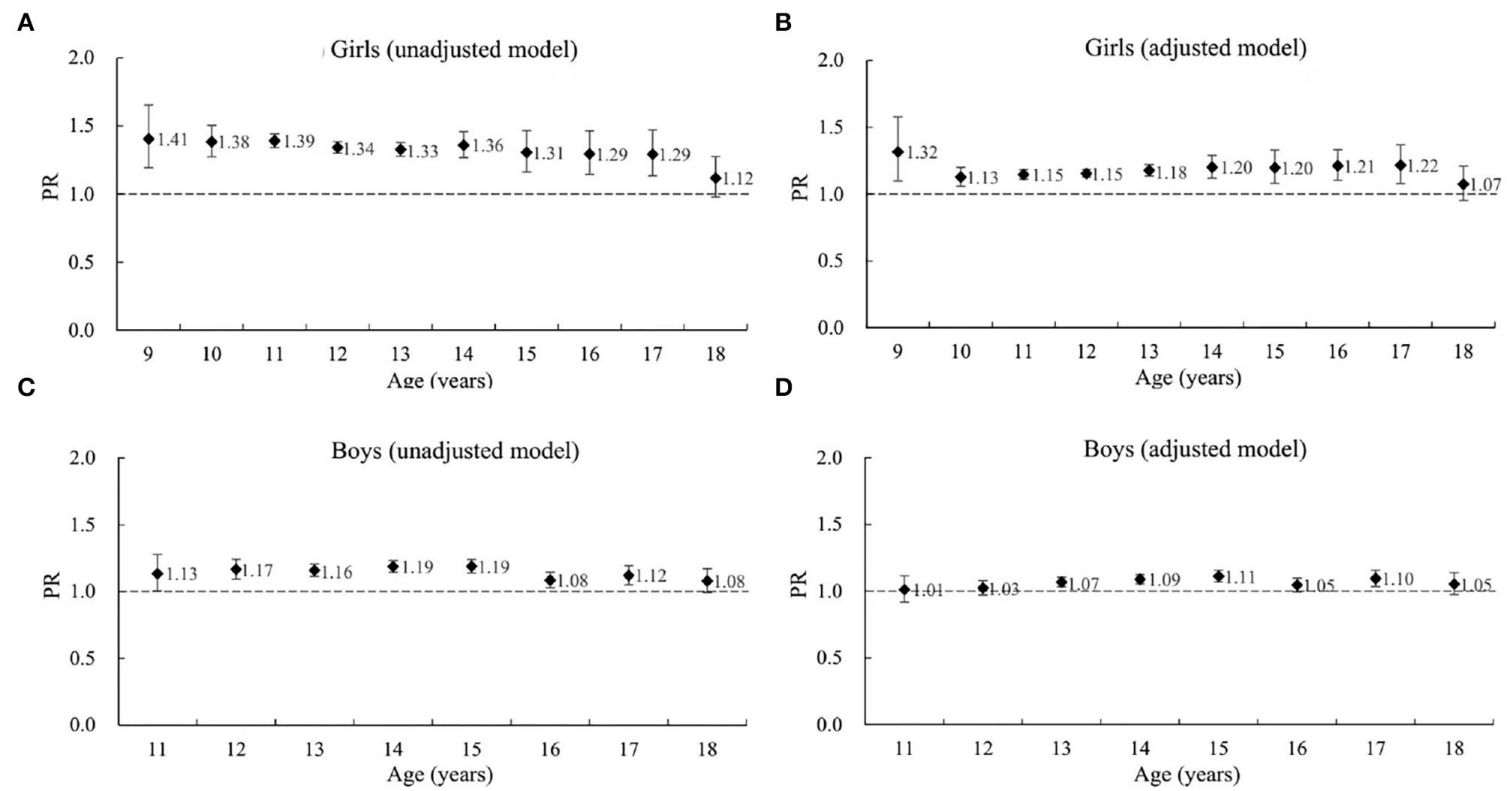
The association between myopia and puberty (menarche for girls and spermarche for boys) status by age in the 1995, 2000, 2005, 2010, and 2014 CNSSCH. **(A)** Girls (unadjusted model). **(B)** Girls (adjusted model). **(C)** Boys (unadjusted model). **(D)** Boys (adjusted model). PR, prevalence ratio, represents the relative risk of myopia for post-menarche girls (or post-spermarche boys) compared to pre-menarche girls (or pre-spermarche boys). CNSSCH, Chinese National Survey on Students' Constitute and Health. The total sample sizes for girls and boys were 429,814 and 332,161, respectively. The unadjusted model only adjusted for the cluster effect of school. The adjusted model additionally adjusted for survey year, urban–rural location, regional socioeconomic status (SES) within province and provincial GDP per capita. The error bars represent 95% confidence intervals (CI).

As shown in Figure 1, post-menarche girls aged 9–17 had 29–41% (PRs ranged from 1.29 to 1.41, all *p* < 0.05) higher risk of being myopic than the pre-menarche girls at the same age. After adjusting for demographic and socioeconomic factors, the PRs consistently reduced to 1.13–1.32 in girls aged 9–17, but all remained statistically significant (*p* < 0.05). The result in 18-year-olds was slightly different, mainly due to the small sample of pre-menarche girls at 18 years of age, as shown in Table 1.

Similarly, unadjusted regression results in boys showed that post-spermarche boys aged 11 to 18 had an 8–19% (PRs ranged from 1.08 to 1.19) higher risk of being myopic than pre-spermarche boys at the same age. All PRs were statistically significant (higher than 1, *p* < 0.05) except in 18-year-olds, where the pre-spermarche sample is small. When further adjusted for demographic and socioeconomic factors, PRs reduced to 1.01–1.11 in the 8 age groups, and only the results of 13–15 and 17-year-olds remained statistically significant (*p* < 0.05).

Table 2 shows the basic characteristics of boys and girls from the paired sample. The median age of post-spermarche boys was only 3 days older than pre-spermarche boys, and the post-menarche girls were only 24 days older than pre-menarche girls (Table 2). Meanwhile, the distribution of pre- and post-spermarche/menarche exact ages was largely overlapping (Supplementary Figure 1), and thus, the residual confounding effect of age in paired sample analyses tended to be minimal.

**Table 2:** The basic characteristics of included pre- and post- menarche/spermarche subjects matched by sex, age, and school from the 2014 CNSSCH, *n* (%).

	**Boys**			**Girls**		
	**Pre-**	**Post-**	***p*-value**	**Pre-**	**Post-**	***p*-value**
**Sample size**	5,641 (100.0)	5,641 (100.0)		6,151 (100.0)	6,151 (100.0)	
**Myopia**			0.26			<0.001
Non-myopia	1,953 (34.6)	1,895 (33.6)		2,336 (38.0)	2,077 (33.8)	
Myopia	3,688 (65.4)	3,746 (66.4)		3,815 (62.0)	4,074 (66.2)	
**Age group**			1.00			1.00
9–10y	0 (0.0)	0 (0.0)		433(7.0)	433(7.0)	
11–12y	1,007 (17.9)	1,007 (17.9)		4,376(71.1)	4,376(71.1)	
13–15y	4,126 (73.1)	4,126 (73.1)		1,312(21.3)	1,312(21.3)	
16–18y	508 (9.0)	508 (9.0)		30(0.5)	30(0.5)	
**Age in days (×100), median(P25, P75)**	51.2 (48.2, 54.2)	51.2 (48.6, 54.4)	0.00	44.5 (42.3, 46.9)	44.8 (42.7, 47.1)	<0.001
**Urban–rural location**			1.00			1.00
Urban	2,884 (51.1)	2,884 (51.1)		3,208 (52.2)	3,208 (52.2)	
Rural	2,757 (48.9)	2,757 (48.9)		2,943 (47.8)	2,943 (47.8)	
**Regional SES within province**			1.00			1.00
Upper	2,026 (35.9)	2,026 (35.9)		2,112 (34.3)	2,112 (34.3)	
Moderate	1,801 (31.9)	1,801 (31.9)		1,998 (32.5)	1,998 (32.5)	
Low	1,814 (32.2)	1,814 (32.2)		2,041 (33.2)	2,041 (33.2)	
**Weekend outdoor activity**			0.16			<0.001
Not in top 3	1,562 (27.7)	1,510 (26.8)		1,610 (26.2)	1,832 (29.8)	
In top 3	4,079 (72.3)	4,131 (73.2)		4,541 (73.8)	4,319 (70.2)	
**Weekend study activity**			0.05			0.44
Not in top 3	698 (12.4)	709 (12.6)		445 (7.2)	468 (7.6)	
In top 3	4,943 (87.6)	4,932 (87.4)		5,706 (92.8)	5,683 (92.4)	
**Physical activity time per day**			0.14			0.31
<30 min	1,211 (21.5)	1,161 (20.6)		1,053 (17.1)	1,104 (17.9)	
30–60 min	2,910 (51.6)	2,870 (50.9)		3,340 (54.3)	3,262 (53.0)	
≥60 min	1,520 (26.9)	1,610 (28.5)		1,758 (28.6)	1,785 (29.0)	
**Homework time per day**			0.40			0.06
<1 h	1,767 (31.3)	1,714 (30.4)		2,719 (44.2)	2,749 (44.7)	
1–2 h	2,218 (39.3)	2,212 (39.2)		2,351 (38.2)	2,241 (36.4)	
≥2 h	1,656 (29.4)	1,715 (30.4)		1,081 (17.6)	1,161 (18.9)	
**Self-report study pressure**			0.14			0.3
Heavy or very heavy	1,996 (35.4)	2,073 (36.7)		1,045 (17.0)	1,001 (16.3)	
So-so or not heavy	3,645 (64.6)	3,568 (63.3)		5,106 (83.0)	5,150 (83.7)	
**Near screen time per day**			0.05			<0.001
0–0.5 h	2,597 (46.0)	2,504 (44.4)		3,711 (60.3)	3,343 (54.3)	
0.5–1 h	1,342 (23.8)	1,314 (23.3)		1,400 (22.8)	1,506 (24.5)	
≥1 h	1,702 (30.2)	1,823 (32.3)		1,040 (16.9)	1,302 (21.2)	
**Sleep duration per day**			0.07			<0.001
<7 h	1,694 (30.0)	1,807 (32.0)		786(12.8)	998(16.2)	
7–8 h	1,951 (34.6)	1,913 (33.9)		1,656 (26.9)	1,861 (30.3)	
≥8 h	1,996 (35.4)	1,921 (34.1)		3,709 (60.3)	3,292 (53.5)	

*CNSSCH, Chinese National Survey on Students' Constitute and Health. Near screen time refers to time spent using a computer, cellphone, tablet, playing video games or reading e-books. All p-values were obtained from chi-square tests except when testing the difference in age in days x 100 (in which a rank sum test was used). “in top 3” and “not in top 3” refers to whether the outdoor (or study) activity is a top 3 choice that the participant usually does on weekends. Because the samples displayed in this table were selected matched samples, rather than the original survey sample, it is inappropriate to use this table to compare the characteristics between boys and girls. To compare boys and girls, please refer to Supplementary Table 3*.

In the paired sample, post-menarche girls had significantly higher myopia prevalence (66.2 vs. 62.0%), less weekend outdoor activity, more near screen time, and shorter sleep duration than pre-menarche girls (*p* < 0.05). No significant differences were seen between the two groups in weekend study activity, daily physical activity time, daily homework time, and self-reported study pressure (*p* > 0.05). Post-spermarche boys had significantly longer near screen time than pre-spermarche boys (*p* < 0.05), but there were no significant differences in myopia prevalence (66.4 vs. 65.4%) or in the six other measured behaviors (Table 2).

Based on the paired sample, post-menarche girls have a 7% (PR = 1.07, 95% CI:1.04–1.10) higher risk of being myopic than pre-menarche girls. This association changed very less when stratified by or adjusted for the seven behaviors (Table 3). However, the association between myopia and spermarche in boys' paired samples was non-significant (unadjusted PR = 1.02, 95% CI:0.99–1.04) and remained non-significant after being stratified by or adjusted for the seven measured behaviors (Table 4).

**Table 3:** The association between menarche status and myopia in girls from the paired sample in the 2014 CNSSCH, stratified by behavioral factors.

	**Sample**	**No. with**	**Prevalence**	***P* for**
	**size**	**myopia**	**ratio (95% CI)**	difference*
**Weekend outdoor activity**				
Not in top 3	3,442	2,178	1.09 (1.04, 1.15)	Ref
In top 3	8,860	5,711	1.06 (1.03, 1.09)	0.267
**Weekend study activity**				
Not in top 3	913	514	1.03 (0.91, 1.16)	Ref
In top 3	11,389	7,375	1.07 (1.04, 1.10)	0.499
**Physical activity time per day**				
<30 min	2,157	1,379	1.09 (1.03, 1.17)	Ref
30–60 min	6,602	4,140	1.06 (1.02, 1.10)	0.754
≥60 min	3,543	2,370	1.07 (1.02, 1.11)	0.497
**Homework time per day**				
<1 h	5,468	3,334	1.08 (1.03, 1.13)	Ref
1–2 h	4,592	3,009	1.06 (1.01, 1.10)	0.498
≥2 h	2,242	1,546	1.07 (1.01, 1.13)	0.798
**Self-reported study pressure**				
Heavy or very heavy	2,046	1,272	1.08 (1.01, 1.15)	Ref
So-so or not heavy	10,256	6,617	1.07 (1.04, 1.10)	0.771
**Near screen time per day**				
0–0.5 h	7,054	4,592	1.06 (1.02, 1.09)	Ref
0.5–1 h	2,906	1,873	1.12 (1.06, 1.18)	0.089
≥1 h	2,342	1,424	1.05 (0.98, 1.13)	0.836
**Sleep duration per day**				
<7 h	1,784	1,147	1.11 (1.03, 1.19)	Ref
7–8 h	3,517	2,360	1.08 (1.03, 1.13)	0.538
≥8 h	7,001	4,382	1.05 (1.01, 1.08)	0.174
**Overall (unadjusted model)**	12,302	7,889	1.07 (1.04, 1.10)	—
**Overall (adjusted model)**	12,302	7,889	1.07 (1.04, 1.10)	—

*CNSSCH, Chinese National Survey on Students' Constitute and Health. Near screen time refers to time spent using a computer, cellphone, tablet, playing video games or reading e-books. In stratified analyses by behavioral factors, an unadjusted model was used to estimate prevalence ratios and their 95% CI. The unadjusted model only controlled the cluster effect of school, while the adjusted model further controlled all behavioral factors in this table. “in top 3” and “not in top 3” refers to whether the outdoor (or study) activity is a top 3 choice that the participant usually does on weekends*.

**Table 4:** The association between myopia and spermarche status in boys from the paired sample in the 2014 CNSSCH, stratified by behavioral factors.

	**Sample**	**No. with**	**Prevalence**	***P* for**
	**size**	**myopia**	**ratio (95% CI)**	difference*
**Weekend outdoor activity**				
Not in top 3	3,072	2,021	1.00 (0.95, 1.05)	Ref
In top3	8,210	5,413	1.02 (0.99, 1.05)	0.496
**Weekend study activity**				
Not in top 3	1,407	780	1.06 (0.96, 1.16)	Ref
In top 3	9,875	6,654	1.03 (0.93, 1.13)	0.379
**Physical activity time per day**				
<30 min	2,372	1,539	1.04 (0.98, 1.11)	Ref
30–60 min	5,780	3,818	1.01 (0.97, 1.04)	0.221
≥60 min	3130	2,077	1.01 (0.97, 1.06)	0.256
**Homework time per day**				
<1 h	3,481	2,007	1.02 (0.97, 1.08)	Ref
1–2 h	4,430,	2,967	1.01 (0.98, 1.05)	0.789
≥2 h	3371	2,460	1.00 (0.97, 1.04)	0.560
**Self-report study pressure**				
Heavy or very heavy	4,069	2,653	1.02 (0.97, 1.07)	Ref
So-so or not heavy	7,213	4,781	1.02 (0.98, 1.05)	0.945
**Near screen time per day**				
0–0.5 h	5,101	3,493	1.05 (1.01, 1.09)	Ref
0.5–1 h	2,656	1,745	0.99 (0.94, 1.05)	0.119
≥1 h	3,525	2,196	0.99 (0.95, 1.04)	0.087
**Sleep duration per day**				
<7 h	3,501	2,407	1.03 (0.99, 1.08)	Ref
7–8 h	3,864	2,561	1.01 (0.96, 1.05)	0.652
≥8 h	3,917	2,466	1.00 (0.96, 1.05)	0.738
**Overall (unadjusted model)**	11,282	7,434	1.02 (0.99, 1.04)	—
**Overall (adjusted model)**	11,282	7,434	1.01 (0.99, 1.04)	—

CNSSCH, Chinese National Survey on Students' Constitute and Health. Near screen time refers to time spent using a computer, cellphone, tablet, playing video games or reading e-books. In stratified analyses by behavioral factors, an unadjusted model was used to estimate the prevalence ratios and their 95% CI. The unadjusted model only controlled for the cluster effect of school, while the adjusted model further controlled all behavioral factors in this table. ^*^ estimated by adding an interaction term to the model. “in top 3” and “not in top 3” refers to whether the outdoor (or study) activity is a top 3 choice that the participant usually does on weekends.

### The Sex Disparity in Myopia

The prevalence of myopia was consistently higher in girls than in boys regardless of age and survey year. Interestingly, in all survey years, the sex differences in myopia prevalence first went up after 9 years of age and then went down after reaching the highest values at 13–15-year-olds. These changes seemed to be in line with the changing pattern of sex disparity in puberty status (Supplementary Table 2).

**Figure 2:**
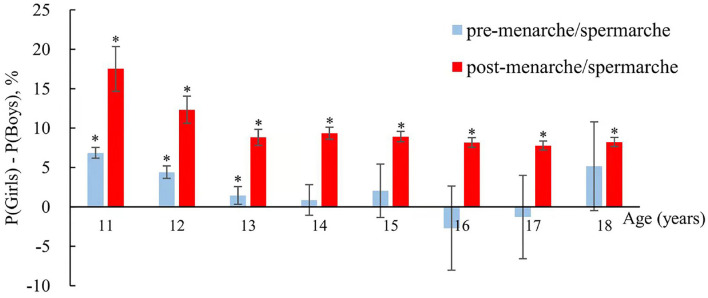
Sex disparity of myopia prevalence among boys and girls aged 11–18 years in the 1995, 2000, 2005, 2010, and 2014 CNSSCH, stratified by age and menarche/spermarche status. P (Girls) and P (Boys) represent the prevalence of myopia in boys and girls, respectively. * represents the statistical significance of sex differences in myopia prevalence.

The sex disparity in myopia was influenced by puberty status. Among post-spermarche/menarche adolescents, girls were 7.8–17.5%points higher in myopia prevalence than boys aged 11–18-year-olds (*p* < 0.05). However, among pre-spermarche/menarche adolescents, the sex differences ranged from −2.7–6.9% points, only reaching significance in 11–13-year-olds (Figure 2).

**Figure 3:**
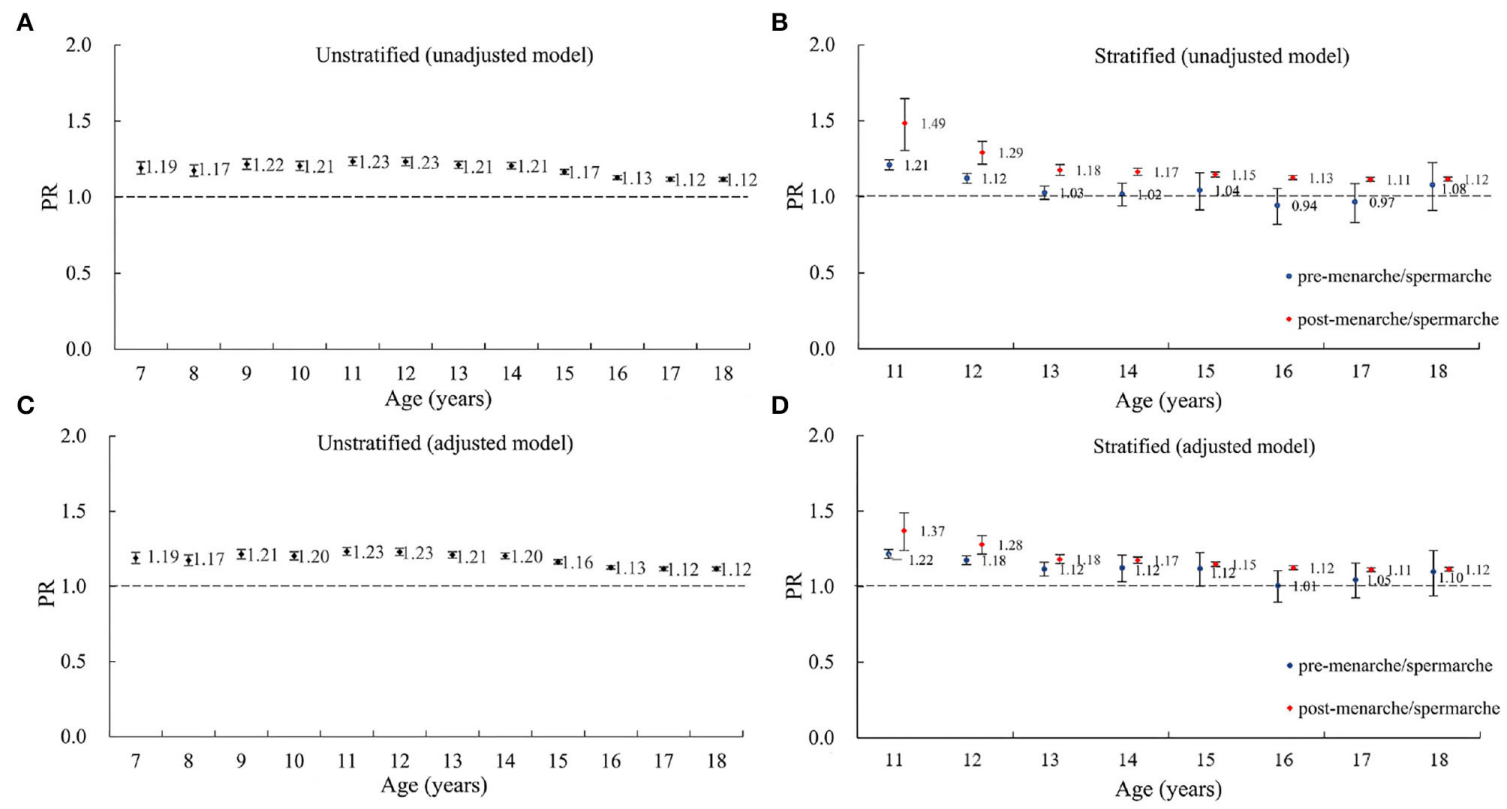
The sex disparity in myopia prevalence estimated by regression models among boys and girls aged 7–18 in the 1995, 2000, 2005, 2010, and 2014 CNSSCH, stratified or unstratified by puberty status. **(A)** Unstratified (unadjusted model). **(B)** Stratified (unadjusted model). **(C)** Unstratified (adjusted model), **(D)** Stratified (adjusted model). PR, prevalence ratio, represents the relative risk of myopia for girls compared to boys. CNSSCH, Chinese National Survey on Students' Constitute and Health. The unadjusted model only adjusted for the cluster effect of school. The adjusted model additionally adjusted for survey year, urban–rural location, regional socioeconomic status (SES) within province, and provincial GDP per capita. The error bars represent 95% confidence intervals (CI).

Figure 3 shows that girls had a 12–23% (PRs ranged from 1.12 to 1.23, all *p* < 0.05) higher risk of myopia than boys at the same age, from 7 to 18 years in both the unadjusted and adjusted models. The post-menarche girls had 11–49% (PRs ranged from 1.11 to 1.49, all *p* < 0.05) higher risk of myopia than post-spermarche boys. However, for pre-spermarche/menarche adolescents aged 11–18 years, girls only had a 1–22% higher risk of myopia than boys.

### The Role of Puberty in Explaining the Sex Disparity in Myopia

As shown in Supplementary Table 2, the biggest difference between sex was puberty status. The median age at menarche was 12.2 years (95%CI: 12.0–12.4), which is 1.6 years earlier than the median age at spermarche (13.8, 95%CI:13.5–14.0). Girls showed less weekend outdoor activity, more weekend study activity, shorter daily physical activity time, shorter sleep duration, and longer homework time; boys were engaged in longer near screen time and reported heavier study pressure (all *p* < 0.001) (Supplementary Table 3).

According to the *basic model 1*, after adjusting for puberty status, the PR for sex disparity decreased from 1.122 to 1.102, indicating that 16.71% [PEMR = (1.122–1.102)/(1.122–1)] of the sex disparity in myopia could be explained by puberty status. In comparison, the seven measured behaviors only explained 0.19–4.78% of the sex disparity in myopia, altogether equaling 11.14%. When fully adjusting for the seven behaviors (*basic model 2*), puberty explained 16.75% of the sex disparity in myopia (Table 5). Using adolescents aged 11–18 years in the 1995, 2000, 2005, and 2010 CNSSCH to repeat the mediation analyses provided consistent results: across years, puberty explained 15.86–21.97% of the sex disparity in myopia after controlling for demographic and socioeconomic factors (Supplementary Table 4).

**Table 5:** The proportion of sex disparity in myopia explained by puberty and behavioral factors, in boys and girls 11 to 18 years of age in the 2014 CNSSCH. sample size = 125,466).

	**Sex (girls vs. boys)**			**PERM**
	**β**	**PR (95%CI)**	**P**	
Unadjusted model	0.11	1.118 (1.105, 1.132)	<0.001	NA
Basic model 1	0.12	1.122 (1.110, 1.135)	<0.001	NA
Additionally adjusted models				
Puberty	0.10	1.102 (1.090, 1.114)	<0.001	16.71%
Weekend outdoor activity	0.12	1.122 (1.109, 1.134)	<0.001	0.61%
Weekend study activity	0.11	1.116 (1.105, 1.129)	<0.001	4.78%
Physical activity time per day	0.11	1.121 (1.109, 1.134)	<0.001	0.94%
Homework time per day	0.11	1.117 (1.105, 1.129)	<0.001	4.30%
Self-report study pressure	0.12	1.122 (1.110, 1.135)	<0.001	0.19%
Near screen time per day	0.11	1.120 (1.108, 1.133)	<0.001	1.68%
Sleep duration per day	0.11	1.121 (1.108, 1.133)	<0.001	1.41%
All seven behavioral factors (Basic model 2)	0.10	1.109 (1.097, 1.120)	<0.001	11.14%
Basic model 2 + puberty	0.09	1.090 (1.079, 1.102)	<0.001	16.75%

*PR, prevalence ratio. PERM, percentage of excess risk mediated. NA, not applicable. In the basic model, we adjusted for exact age, urban–rural location, regional SES within province, fixed effect of province, and the cluster effect of school*.

## Discussion

Menarche is a major milestone of female puberty (17), just as spermarche is for boys. We found that menarche was associated with a 7% higher risk of myopia among girls, but the association between spermarche and myopia in boys was smaller and non-significant. The sex disparity in myopia was consistent across 7–18-year-olds in all 5 surveyed years. Interestingly, the sex disparity in myopia was stronger and significant in post-menarche/spermarche adolescents, but smaller or non-significant in pre-menarche/spermarche adolescents. Over 16% of the sex disparity in myopia could be explained by girls' earlier puberty, compared to ~11% explained by several other behavioral factors together.

The mechanism underlying the association between myopia and puberty is unclear. It is plausible that when the body grows rapidly during puberty, the axial length of the eyes also grows faster, so puberty could create a risk for myopia onset due to axial growth. The mechanisms that link puberty development and axial growth are unknown, but Lyu believes that increased estrogen after menarche could explain the association (12). Although two case–control studies found that serum estrogen was not higher in myopic girls compared with non-myopic girls, the two studies had several major flaws, including a small sample size and not controlling for critical confounders (e.g., age, outdoor time) (18, 19). Insulin-like growth factor-1 (IGF-1) is another, perhaps more convincing mediator. Serum IGF-1 level grows rapidly after menarche/spermarche (20, 21), and it could accelerate axial elongation in eyes (22) according to experimental studies in chicks (23, 24) and genetic studies (25). More human-based evidence is needed to test whether IGF-1 is an underlying mechanism driving axial growth during puberty.

The sex difference in the association between myopia and puberty may be explained by the differences in physiological and behavioral changes during puberty. Physiological changes could include hormone changes during puberty (e.g., androgen in boys vs. estrogen in girls), although the role played by hormones in myopia onset remains unknown (18, 19, 26). As for behavioral changes, menarche and spermarche, the milestones of sexual maturity (27), may make adolescents more concerned about appearance. In the perspective of Asian countries, white skin is a key component of attractiveness in girls. Thus, post-menarche girls may reduce outdoor activity to avoid being tanned and sweating from activities. This was supported by our finding that post-menarche girls were less active in weekend outdoor activities than pre-menarche girls, whereas no such difference was found between pre- and post-spermarche boys.

Sex disparity in myopia is a widespread phenomenon, especially in eastern Asia (2). Our results further support this conclusion. Traditional Chinese culture typically requires women to be quiet and men to be active (28), so previous studies have suggested that the sex disparity in myopia may be due to behavioral differences in outdoor activity and near work (activities requiring near focus). Also, the interest Chinese girls have in paler skin may make them avoid outdoor activities that are known to protect against myopia (29, 30). Girls generally study harder than boys and have longer homework time (29), predisposing them to myopia. However, the seven measured behaviors in our mediation analyses could each only explain 0.19–4.78% of the sex disparity in myopia (cumulatively explaining 11.14%). In contrast, puberty could explain more than 16% of the sex disparity. The mediation effect of puberty had two foundations. One was the association between myopia and puberty, as we discussed above. The other was the difference in puberty timing, where boys generally start puberty 1–2 years later than girls (5, 6). Compared to the behavioral factors described above, puberty is a better explanation of the sex disparity in adolescent myopia in other countries where the sex difference in those behaviors (e.g., outdoor activity, white skin preference, and homework time) may not exist or be reversed. Admittedly, puberty cannot explain the sex disparity in 7–9-year-olds, and there is still a large proportion of the sex disparity to be explained by other factors, such as opsin genetics. The human retina contains three types of cone photoreceptors, which are sensitive to long (L), middle (M), or short (S) wavelengths of light. Recent studies indicate that the L:M cone ratio, combined with L and/or M opsin exon 3 haplotypes at chromosome location Xq28, cause minor splicing defects that could increase myopia susceptibility. Because girls have two X-chromosomes, they are two times as likely to carry a cone opsin polymorphism, potentially making them more likely to develop myopia (31, 32).

This study has two public health implications. First, girls' earlier puberty contributes significantly to their higher prevalence of myopia than boys. This will put them at a higher risk of developing larger-grade myopia at an earlier age and therefore increase their risk of developing secondary ocular pathology (33–35). For these reasons, early interventions for preventing myopia onset might be more important in girls. Because our study did not analyze refraction and axial length data, it remains unclear whether earlier puberty could lead to a higher degree of myopia in girls. Until the evidence becomes clear, it is suggested to use early prevention and intervention methods against myopia irrespective of sex. The second public health implication is that menarche status seems to be an independent risk factor of myopia in girls. Thus, the early phases of puberty may be a sensitive period to control myopia in girls, and preventive strategies such as vision screening and increasing outdoor activity should be targeted to girls during this period.

The results of our study are consistent with findings from India (12) and South Korea (13), but our study uniquely minimizes recall bias because menarche statuses and covariates were gathered in participants' adolescence rather than in their adulthood. Although our findings differ from an analysis from Singapore (8), our study benefited from large, nationwide samples, minimizing selection bias and the risk of false-negative results due to low statistical power. Moreover, our study detected a small association between myopia and spermarche that was not observed in the previous studies.

Our study had several limitations that should be acknowledged. First, although our definition of myopia is not widely used, it is useful in the context of Chinese schools, where nearly 90% of vision impairment is due to uncorrected myopia. The increased statistical power of the large, uniformly-collected dataset justifies our use of unaided visual acuity as a surrogate for myopia (36). The convenience and accuracy of using unaided visual acuity assessment combined with simple subjective refraction has led the Chinese government to advocate its use for myopia screening in school children (37). Also, our validation trial has shown this method achieved high accuracy (refer to Supplementary Material) despite some misclassifications. The misclassifications tended to be non-differential (i.e., not related to sex and puberty) because the VA measurement followed a standard procedure independent of the participant's sex and puberty status. Additionally, the VA procedure had been used for over 30 years in CNSSCH and was implemented by well-trained examiners. If anything, non-differential misclassifications would likely attenuate the effects of puberty status and sex on myopia. Second, our study was not a cohort study, so the causal relationship between outcome and exposure cannot be established with certainty. While prospective studies could help to establish causality, it is unlikely that myopia causes early puberty. Third, CNSSCH questionnaires did not gather longitudinal information before or at puberty onset for each child, so important factors such as hormonal changes in puberty and social development were not considered. Plus, the questionnaires could not precisely measure behavioral factors such as near work, physical activity, and outdoor time, which could lead to residual confounding. For example, daily outdoor time was estimated by weekend outdoor activity frequency and daily physical activity time (given that most school gyms in China are outdoors). Despite this limitation and the lack of hormone biomarkers of sexual development, our findings provide a solid platform that can inform health and educational policies. Also, we did not measure actual refraction and axial length, and thus, our study can only provide implications on the impacts of puberty and sex on the prevalence of myopia, but not on the degree of myopia. The Chinese government is planning to include these measures in the future national monitoring system (37). Further, future iterations of the CNSSCH may be useful for assessing the regional and national impacts of evolving policies. Finally, while puberty and age are closely related and associated with the development of myopia, our matched sample analysis allowed us to precisely separate these two highly correlated factors.

Previous evidence suggests that physical activity might affect the timing of puberty onset in girls (33), potentially confounding menarche–myopia associations. Inaccurate physical activity measurements could also cause residual confounding. Fortunately, the overall confounding effect of the behaviors analyzed in this study was minimal. Additionally, although the timing of puberty onset in girls can also be affected by the age of parental puberty, body weight, high animal protein intake, and family stressors (38), these factors had a weak link with myopia (39) and are unlikely to confound or bias menarche–myopia associations.

In conclusion, puberty status among Chinese adolescents might be an independent risk factor for myopia in girls but not boys, suggesting early and mid-puberty may be a sensitive period for girls' myopia prevention. Earlier puberty in girls explained a significant proportion of the sex disparity in myopia prevalence, but detailing the public health implications of this finding requires further longitudinal studies with more accurate measures of myopia and puberty status.

## Data Availability Statement

All individual (de-identified) participant data collected in the surveys are accessible upon request. Researchers who are interested in using the data should contact Prof. Jun Ma (majunt@bjmu.edu.cn) and Prof. Yi Song (songyi@bjmu.edu.cn) with a study protocol and statistical analysis plan. There will be an assessment proposed by an independent review committee. Once approved by the committee, the researcher can access the data. An agreement on the use of the data may also needed.

## Ethics Statement

The studies involving human participants were reviewed and approved by the Medical Research Ethics Committee of Peking University Health Science Center (IRB00001052-18002) following the Declaration of Helsinki. Written informed consent to participate in this study was provided by the participants' legal guardian/next of kin.

## Author Contributions

RX and YS conceived the study and its design. RX and PZ performed data analysis, and drafted the initial manuscript. RS, CJ, XX, DL, and YS modified the manuscript. RS, CJ, JM, and YS refined the data analysis plans and interpretation of the findings. JM, YS, YD, DL, and XX contributed to data collection. YS contributed to manuscript preparation and had full access to all aspects of the research and writing process as well as primary responsibility for the final content. All authors contributed to the article and approved the submitted version.

## Funding

This study was supported by the China Medical Board (Grant #21-434 to YS) and Capital's Funds for Health Improvement and Research (2022-1G-4251 to YS). RX was funded by the China Scholarship Council (201806010405) to study in Monash University. CJ was supported by a postgraduate scholarship from the Australian Commonwealth Government. The funding bodies did not have a role in the design of the study and collection, analysis, data interpretation, or writing of this manuscript.

## Conflict of Interest

The authors declare that the research was conducted in the absence of any commercial or financial relationships that could be construed as a potential conflict of interest.

## Publisher's Note

All claims expressed in this article are solely those of the authors and do not necessarily represent those of their affiliated organizations, or those of the publisher, the editors and the reviewers. Any product that may be evaluated in this article, or claim that may be made by its manufacturer, is not guaranteed or endorsed by the publisher.
