# Supplementary material for: Sex Disparity in Myopia Explained by Puberty Among Chinese Adolescents From 1995 to 2014: A Nationwide Cross-Sectional Study
Source: Front Public Health. 2022 May 30;10:833960. doi: 10.3389/fpubh.2022.833960 (PMC9196902; doi:10.3389/fpubh.2022.833960)
Supplement: Supplementary file 1 [file Data_Sheet_1.docx]

**Part 1: The validation trial of the definition of myopia in Chinese National Survey on Students’ Constitue and Health (CNSSCH)**

In 2012, our collaborators in Anhui Medical University conducted a validation trial to test the accuracy of the definition of myopia that have been used in CNSSCN since 1985. The results was not published in English, but the details of the validation trial in Chinese could be found in Pei’s master’s thesis (accessible at <http://www.cnki.net/>)^1^. Here are some primary information of the validation trial:

**Participants:** Students at grade 1-8 were recruited from 18 primary and secondary schools in Shenyang by multi-stage cluster sampling procedure. All included students and their parents signed the consent form. Students with strabismus, amblyopia and other significant eye diseases were excluded. Finally, a total of 1620 students (50.0% female) with signed consent form were included in the trial. The project was approved by the ethics committee of Anhui Medical University (grant No. 2013001).

**Measurements:** For all participants (both eyes), the myopic status was measured by both the method descripted in our manuscript and cycloplegic refraction. The gold standard of myopia was defined as spherical equivalent cycloplegic refractive error ≤-0.50 D for at least one eye.

**Funding source:** This trial was a subproject of the ‘Students Major Disease Prevention & Control Technology and Its Development and Application’ (1147 project, Grant No.201202010), funded by the research special fund for public welfare industry of health of the Ministry of Health of China. The principal investigator of the 1147 project is Prof. Jun Ma, one of our co-authors and the head of the Institute of Child and Adolescent Health, Peking University.

**Main results:** see table S1 below.

**Part 2: Mediation analyses**

We performed a two-step mediation analyses according to the method used by Zylbersztejn et al’s^2^ paper published on the *Lancet* in 2018. Assuming causality, the framework or hypothesis could be represented by diagram below:


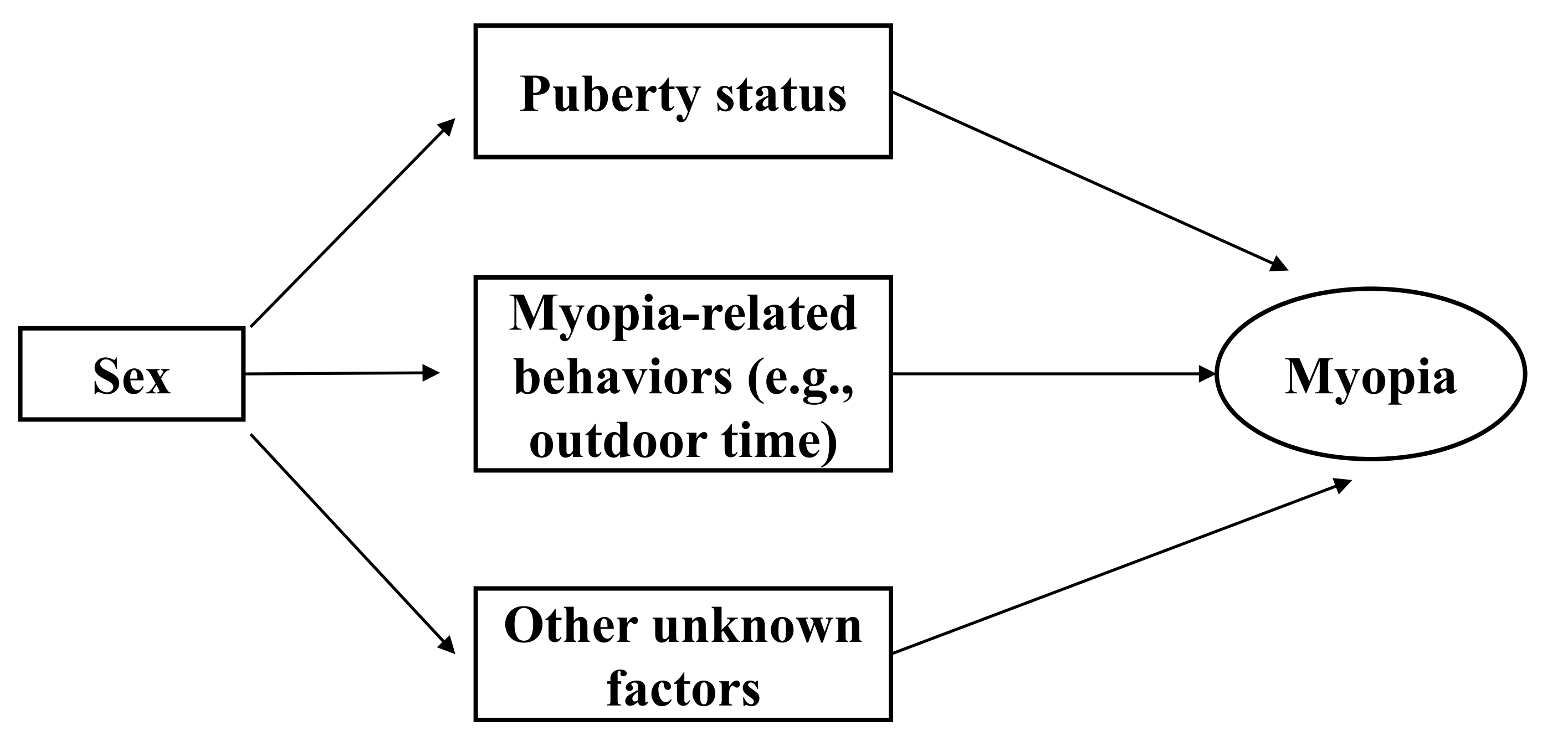


In the first step, we used a basic model where myopia is the dicrotous outcome variable while sex is a categorical independent variable. We adjusted for demographic characteristics (age, urban-rural location, regional SES within province, fixed effect of province and the cluster effect of school) in this basic model (basic model 1), from which we obtained the prevalence ratio (PR) representing the relative risk of myopia in girls compared to boys, recorded as PR _sex basic_.

In the second step, we added one independent variable (puberty status or behavioral factors) to the basic model and obtained the PR _sex adjusted_. Then we calculated percentage of excess risk mediated (PERM) for each variable, taking puberty status as an example:

PERM _puberty status_ =100%*(PR _sex basic_- PR _sex adjusted for puberty status_)/(PR _sex basic_ -1).

The PERM _puberty status_ can be interpreted as the proportion of sex disparity explained by puberty status. We established another basic model (basic model 2) that additionally adjusted for all behavioral factors we measured excepted puberty status. Then similar two-step mediation analyses used to estimate the PERM _puberty status_ that has excluded the impact of behavioral factors.

**Part 3: Other supplemental figures and tables**


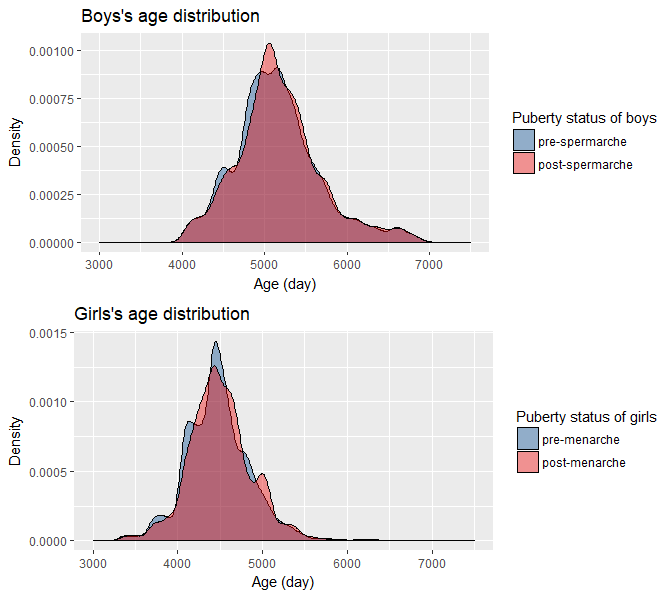


**Figure S1.** The distribution of exact age of pre- and post-spermarche/menarche boys/girls of the paired sample selected from the 2014 CNSSCH.

Note: CNSSCH, Chinese National Survey on Students’ Constitute and Health.

| **Table S2. Comparison of the myopia prevalence between boys and girls aged 7 to 18 years in CNSSCH 1995-2014 [n(%)]** | | | | | | | | | | | | | | | | | | | |
| --- | --- | --- | --- | --- | --- | --- | --- | --- | --- | --- | --- | --- | --- | --- | --- | --- | --- | --- | --- |
|  | **1995** | | |  | **2000** | | |  | **2005** | | |  | **2010** | | |  | **2014** | | |
| **Age(years)** | **Boys** | **Girls** | **P-value** |  | **Boys** | **Girls** | **P-value** |  | **Boys** | **Girls** | **P-value** |  | **Boys** | **Girls** | **P-value** |  | **Boys** | **Girls** | **P-value** |
| 7 | 8191(12.9) | 8188(17.5) | <0.001 |  | 9059(13.9) | 9000(17.1) | <0.001 |  | 9752(18.7) | 9624(22.3) | <0.001 |  | 8972(25.6) | 8969(29.1) | <0.001 |  | 8933(24.6) | 8923(27.8) | <0.001 |
| 8 | 8185(12.0) | 8203(14.6) | <0.001 |  | 9075(13.8) | 9007(16.6) | <0.001 |  | 9725(20.9) | 9608(24.9) | <0.001 |  | 8963(26.8) | 8962(31.8) | <0.001 |  | 8915(28.2) | 8912(31.1) | <0.001 |
| 9 | 8187(14.4) | 8194(18.6) | <0.001 |  | 9006(14.2) | 9001(18.0) | <0.001 |  | 9759(22.3) | 9299(28.4) | <0.001 |  | 8970(31.9) | 8984(38.0) | <0.001 |  | 8933(34.7) | 8954(39.7) | <0.001 |
| 10 | 8746(18.6) | 8759(22.8) | <0.001 |  | 9121(17.5) | 9146(22.7) | <0.001 |  | 9874(28.0) | 9420(35.1) | <0.001 |  | 8964(39.0) | 8972(45.9) | <0.001 |  | 8943(42.6) | 8926(48.5) | <0.001 |
| 11 | 8750(23.0) | 8664(29.6) | <0.001 |  | 8995(20.6) | 8971(28.4) | <0.001 |  | 9869(32.8) | 9370(41.5) | <0.001 |  | 8987(44.9) | 8990(53.4) | <0.001 |  | 8946(48.8) | 8895(57.0) | <0.001 |
| 12 | 8740(28.6) | 8600(37.0) | <0.001 |  | 8998(25.8) | 9019(34.4) | <0.001 |  | 9717(36.3) | 9215(47.1) | <0.001 |  | 8948(49.0) | 8981(57.5) | <0.001 |  | 8889(54.2) | 8885(61.8) | <0.001 |
| 13 | 8741(39.4) | 8555(49.0) | <0.001 |  | 8895(34.1) | 9011(43.1) | <0.001 |  | 9677(43.6) | 9391(54.7) | <0.001 |  | 8960(56.3) | 8966(66.4) | <0.001 |  | 8904(60.1) | 8895(70.0) | <0.001 |
| 14 | 8749(44.9) | 8577(54.9) | <0.001 |  | 8986(42.4) | 8968(52.7) | <0.001 |  | 9666(49.1) | 9292(62.3) | <0.001 |  | 8959(62.3) | 8961(71.2) | <0.001 |  | 8933(63.5) | 8887(75.5) | <0.001 |
| 15 | 8747(50.9) | 8584(60.9) | <0.001 |  | 9018(51.9) | 8952(64.0) | <0.001 |  | 9930(58.7) | 9475(69.9) | <0.001 |  | 8921(66.8) | 8935(75.2) | <0.001 |  | 8916(69.9) | 8918(77.4) | <0.001 |
| 16 | 8682(60.0) | 8518(67.9) | <0.001 |  | 8995(61.7) | 8968(72.0) | <0.001 |  | 9804(66.9) | 9402(75.2) | <0.001 |  | 8936(72.8) | 8871(80.9) | <0.001 |  | 8872(73.4) | 8866(80.1) | <0.001 |
| 17 | 8729(64.1) | 8531(72.1) | <0.001 |  | 8893(67.9) | 8961(77.1) | <0.001 |  | 9790(70.9) | 9371(78.7) | <0.001 |  | 8929(74.8) | 8933(82.7) | <0.001 |  | 8900(74.5) | 8876(81.0) | <0.001 |
| 18 | 8655(64.5) | 8456(73.8) | <0.001 |  | 9088(69.7) | 9167(78.5) | <0.001 |  | 10117(71.0) | 9870(80.1) | <0.001 |  | 8914(75.7) | 8857(82.1) | <0.001 |  | 8507(75.0) | 8450(82.2) | <0.001 |
| Total | 103102(36.4) | 101829(43.4) | <0.001 |  | 108129(36.1) | 108171(43.7) | <0.001 |  | 117680(43.4) | 113337(51.7) | <0.001 |  | 107423(52.1) | 107381(59.5) | <0.001 |  | 106591(54.0) | 106387(60.9) | <0.001 |
| Note: CNSSCH=Chinese National Survey on Students’ Constitute and Health. P-values were obtained from Chi-square test. | | | | | | | | | | | | | | | | | | | |

| **Table S4.** The proportion of sex disparity in myopia explained by puberty status among boys and girls aged 11~18 in CNSSCH 1995 to 2010 | | | | | | | | | | |
| --- | --- | --- | --- | --- | --- | --- | --- | --- | --- | --- |
| survey year | sample size | Unadjusted model | |  | Basic model | |  | Adjusted model | | PEMR |
|  |  | PR (95% CI) | P-value |  | PR (95% CI) | P-value |  | PR (95% CI) | P-value |  |
| 1995 | 137177 | 1.18(1.15, 1.22) | <0.001 |  | 1.18(1.16, 1.21) | <0.001 |  | 1.14(1.12, 1.17) | <0.001 | 21.97% |
| 2000 | 141845 | 1.20(1.18, 1.23) | <0.001 |  | 1.21(1.19, 1.23) | <0.001 |  | 1.16(1.14, 1.18) | <0.001 | 20.56% |
| 2005 | 141916 | 1.18(1.16, 1.20) | <0.001 |  | 1.19(1.17, 1.20) | <0.001 |  | 1.16(1.14, 1.18) | <0.001 | 15.86% |
| 2010 | 131331 | 1.12(1.11, 1.14) | <0.001 |  | 1.14(1.12, 1.15) | <0.001 |  | 1.11(1.10, 1.13) | <0.001 | 15.95% |
| all years combined | 552269 | 1.18(1.16, 1.19) | <0.001 |  | 1.18(1.17, 1.19) | <0.001 |  | 1.14(1.13, 1.15) | <0.001 | 19.86% |
| Note: PR=prevalence ratio. PERM=percentage of excess risk mediated. In the basic model, we adjusted for age, urban-rural location, regional SES within province, fixed effect of province and the cluster effect of school. The adjusted model further adjusted for puberty status in addition to the basic model. | | | | | | | | | | |

**Reference**

1. Pei Chenlu. Studies of vision care related behaviors and methods for myopia screening in Chinese elementary and secondary students. MS thesis. Anhui Medical University, 2014.(in Chinese)

2. Zylbersztejn A, Gilbert R, Hjern A, et al. Child mortality in England compared with Sweden: a birth cohort study. Lancet 2018;391(10134):2008-18.
